# Latent Profile Analysis of Acute Stress Disorder Symptoms and Their Links to Individual Characteristics and Mental Health Among College Students During the Early COVID-19 Pandemic

**DOI:** 10.3390/bs14111020

**Published:** 2024-11-01

**Authors:** Xiaoqi Yan, Bin Gao, Xianghua Cai, Yongsheng Fan, Chong Zhao, Lu Bai

**Affiliations:** 1 School of Education, Shanghai Normal University, Shanghai 200234, China; 2 School of Vocational and Technical Teacher Education, Shanghai Polytechnic University, Shanghai 201209, China; 3 Department of Physical Education, Shanghai University of Medicine & Health Sciences, Shanghai 201318, China; 4 College of Education, Jiangsu University of Technology, Changzhou 212003, China

**Keywords:** acute stress disorder, mental health, latent profile analysis, college students

## Abstract

Objectives: acute stress disorder (ASD) became prevalent among various populations during the COVID-19 pandemic, yet little research has examined the heterogeneity of ASD symptoms among college students. The purpose of this research was to explore subgroups of ASD symptoms using latent profile analysis (LPA) and to explore the predictors and mental health outcomes associated with these profiles. Methods: Using the person-centered method, we recruited 1198 college students (71.7% female) who self-reported their ASD, perceived social support, anxiety, depression, and life satisfaction from two Chinese universities following the COVID-19 outbreak. Results: The LPA results found three ASD symptom severity profiles: low (56.7%), moderate (31.6%), and high (11.7%), particularly characterized by reexperiencing and arousal symptoms. This study found that students in the moderate and high ASD subgroups were more likely to be female, have lower socioeconomic status, belong to minority groups, report lower self-rated health, and perceive less social support compared to those in the low ASD subgroup. Furthermore, compared to the low and moderate ASD subgroups, the high ASD subgroup was linked to elevated anxiety and depression and lower life satisfaction. Conclusions: These findings underscore the significance of identifying specific ASD symptom subgroups to effectively target prevention and intervention efforts.

## 1. Introduction

Acute stress disorder (ASD) is a mental health state induced by stress and trauma, marked by symptoms such as intrusive thoughts, intense anxiety, depersonalization, avoidance behaviors, and heightened arousal, generally manifesting within three days to a month of the traumatic event, which may be transient or could lead to PTSD if symptoms persist for more than a month after the traumatic event [1]. ASD can impair social and occupational functioning, disrupt daily activities, and result in comorbid mental health conditions such as anxiety and depression [2,3]. Furthermore, recent findings highlight an increased risk of suicidal ideation among those with ASD [4]. During the COVID-19 pandemic, ASD became prevalent among various populations, including nurses [5], teachers [6], and civilians [7]. However, previous research on ASD has largely overlooked the university student population. In fact, college students faced numerous challenges across various domains during the COVID-19 pandemic, such as social isolation [8], fears of viral contamination [9], disruptions to their daily routines [10], academic challenges [11], and high employment pressure [12]. These difficulties led to pronounced psychological issues including ASD.

Furthermore, a variable-centered approach has predominated in earlier studies to explore the relationships and mechanisms between ASD and other variables. This approach does not treat individuals holistically. In contrast, person-centered approaches can delineate subgroups of individuals by analyzing within-group data similarities as opposed to between-group differences [13,14], suggesting that a combination of ASD symptoms (e.g., dissociation, reexperiencing, avoidance, and arousal) may manifest uniquely and have distinct consequences when together compared on an individual basis. Therefore, the person-centered method offers a distinct viewpoint on ASD, enhancing the insights gained from the variable-centered method. Up to now, limited research has explored the heterogeneity of ASD symptoms among college students.

### 1.1. Literature Review

Diverse subtypes of ASD symptoms can be identified through person-centered methods [15]. Latent profile analysis (LPA) has been employed in studies of ASD to delineate different symptom profiles across various populations and trauma exposures. These studies frequently find that ASD profiles are mainly categorized by symptom severity, with the number of detected subgroups typically ranging from three to five. For instance, Lenferink et al. [16] used LPA to identify three ASD classes in children exposed to single-incident traumas, noting differences in impairment and symptom severity. Straud et al. [17] also identified three ASD profiles, but in blast-injured military personnel, with the most severe profile exhibiting high hyperarousal and reexperiencing symptoms. Similarly, McKinnon et al. [18] found that a three-profile solution best represented ASD symptoms in trauma-exposed youth, suggesting the need for a more nuanced understanding of ASD in this population. In contrast, Shevlin et al. [19] found four ASD classes in female victims of sexual trauma, with high ASD and intermediate classes showing elevated risks for PTSD. Additionally, Armour and Hansen [15] identified four ASD subtypes in bank robbery victims, including an intrusive subtype influenced by age, social support, and peritraumatic panic. Unlike the aforementioned studies, Hansen et al. [20] identified five ASD subcategories in a diverse sample of trauma survivors, emphasizing a severely symptomatic group marked by elevated avoidance and dissociation levels. These studies collectively underscore the heterogeneity of ASD symptom profiles and the importance of identifying specific subtypes to enhance targeted interventions.

While previous studies offer insights into ASD symptom profiles, their applicability to COVID-19-induced ASD may be limited. In addition, another common limitation across previous studies is the lack of focus on college students who have experienced COVID-19-related trauma. No studies we know of have used LPA to assess the heterogeneous symptom profiles associated with ASD among university students during the acute phase of the COVID-19 outbreak. Such research is crucial for the early detection of ASD symptoms, which may help prevent the progression of COVID-19-related mental health issues and address a significant gap in understanding this pandemic’s impact on university students. Additionally, previous LPA research has not explored predictors such as demographic characteristics and social support, nor the outcomes like anxiety, depression, and life satisfaction associated with different ASD symptom profiles. Furthermore, prior evidence on LPA in ASD has been inconsistent due to variations in sample characteristics and the nature of acute stress scenarios. Most studies have identified both three-profile and four-profile solutions. Consequently, it is essential to identify ASD profiles specific to university students during the COVID-19.

### 1.2. Research Questions and Hypothesis Development

In this study, we aimed to address three primary research questions. First, what types of ASD profiles emerged naturally among undergraduates during COVID-19? Second, can personal factors, such as demographics and perceived social support, predict the likelihood of individuals being classified into specific ASD profiles? Third, are these ASD profiles differently linked to various mental health-related variables, including anxiety, depression, and life satisfaction?

Therefore, the first purpose of this research was to investigate the diversity of ASD profiles among undergraduates shortly after the COVID-19 outbreak. On the basis of the previous literature that identified subgroups with ASD symptoms in various samples [17,18], our hypothesis was that the most appropriate model would be a three- or four-profile solution, with profiles differing mainly by ASD symptom severity. The second goal was to explore the predictors of ASD symptom profiles by evaluating the impacts of individual characteristics on the likelihood of classification into specific ASD profiles. Drawing from previous research indicating that factors such as gender, age, minority status, socioeconomic status (SES), and social support significantly predict ASD symptoms [15,21], we hypothesized that ASD subtypes in college students could be predicted by these individual characteristics and social support. The third objective was to assess the relationship between ASD profiles and potential mental health outcomes. Building on previous research highlighting the strong link between ASD symptoms, anxiety, and depression [2,22,23], it was hypothesized that ASD profiles with higher symptoms would be linked to increased anxiety and depression and lower life satisfaction.

**Figure 1:**
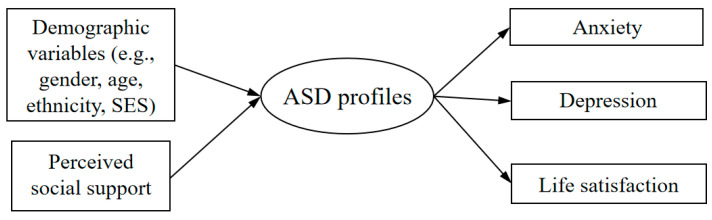
Conceptual framework for this study. Note. SES = socioeconomic status, ASD = acute stress disorder.

We constructed a conceptual framework (see Figure 1) to illustrate the relationships examined in this study. This framework shows how demographic indicators (e.g., gender, age, ethnicity, socioeconomic status) and perceived social support predict ASD profiles, which are then analyzed for their associations with anxiety, depression, and life satisfaction.

## 2. Materials and Methods

### 2.1. Participants and Procedure

This study’s participants were individuals who potentially had COVID-19 and had not been diagnosed. This means that the participants may have been at risk of infection, either due to symptoms or exposure, but had not yet undergone diagnostic testing or received confirmation of infection at the time of their participation in this study. A total of 1198 subjects were selected through convenience sampling from two public universities located in Guangxi and Jiangxi provinces in China. The sample included 339 male students (28.3%) and 859 female students (71.7%). The distribution by academic year was 217 freshmen, 417 sophomores, 349 juniors, and 215 seniors. Among the participants, 852 were Han Chinese (71.1%), and 346 were from ethnic minority groups (such as Zhuang, Miao, Tujia, and other officially recognized minority groups besides the Han majority). The subjects had an average age of 19.62 years (SD = 1.38).

This study employed a cross-sectional design to investigate the heterogeneity of acute stress disorder (ASD) symptoms among college students during the early period of the COVID-19 outbreak in Mainland China, specifically from February to March 2020. All participants were under home quarantine during the survey period. Data were collected using electronic questionnaires distributed by the investigators via social media platforms, including WeChat and Tencent QQ. Participants were informed that completing the questionnaire would take approximately 5–8 min. Informed written consent was obtained as part of the instruction section of the questionnaire before participants commenced the survey. The questionnaire was administered in Chinese, ensuring clarity and understanding for all respondents. Participation in this study was entirely voluntary, and no incentives were provided. Ethical approval for this study was obtained from the Shanghai Normal University Ethics Committee, ensuring that all ethical considerations were met, including participant confidentiality and data protection. This comprehensive approach allowed for a thorough exploration of ASD symptoms in the context of the COVID-19 pandemic.

### 2.2. Measures

#### 2.2.1. Acute Stress Disorder Scale

Symptoms of acute stress disorder were assessed using the acute stress disorder scale [24], consisting of 19 items. The ASDS encompasses four subscales: dissociation, reexperiencing, avoidance, and arousal. Subjects evaluated the degree to which they encountered each symptom since the COVID-19 outbreak on a scale from 1 (not at all) to 5 (very much). This scale has been shown to have good reliability and validity in the Chinese population [25]. In the current sample, the Cronbach’s alpha for the scale was 0.88.

#### 2.2.2. Perceived Social Support Scale

This study utilized the short version of the Perceived Social Support Scale developed by Lin et al. [26]. This instrument comprises 6 items, such as “There is someone I trust who would help me if I needed it.” Cross-cultural research involving participants from the United States, Russia, Germany, and China has confirmed the scale’s strong reliability and validity [26]. Responses are collected using a 7-point Likert scale, spanning from 1 (“strongly disagree”) to 7 (“strongly agree”), where elevated scores reflect a greater perception of social support. In this study, the scale demonstrated a Cronbach’s alpha of 0.84.

#### 2.2.3. The General Anxiety Symptoms Scale (GAD-7)

Anxiety symptoms were assessed using the GAD-7 scale [27], comprising 7 items. This instrument has demonstrated robust reliability and validity among Chinese people [28]. The scale uses a 4-point Likert format, ranging from 0 (not at all) to 3 (nearly every day). Elevated scores reflect higher levels of anxiety symptoms. In the current study, the scale exhibited good internal consistency (Cronbach’s α = 0.89).

#### 2.2.4. The Patient Health Questionnaire (PHQ-9)

The PHQ-9 was employed in this study to evaluate depression [29]. This tool features a unidimensional structure comprising 9 items. The PHQ-9 Chinese version, adapted and verified by Du et al. [30], was employed. Responses are rated on a 4-point scale from 0 (Not at all) to 3 (Almost every day), with elevated scores indicating greater depressive tendencies. The instrument demonstrated good internal consistency (Cronbach’s α = 0.90).

#### 2.2.5. The Satisfaction with Life Scale (SWLS)

The SWLS, initially created by Diener et al. [31] and validated among Chinese college students [32], was used to assess life satisfaction. The tool comprises five items that evaluate subjective judgments on life quality. Participants rate their responses on a 5-point scale (1 = “strongly disagree”, 5 = “strongly agree”). Elevated average scores reflected greater levels of life satisfaction. The Cronbach’s α of this tool was 0.84 in this study.

### 2.3. Data Analysis

Data cleaning, descriptive statistics, and scale reliability analyses were performed using SPSS 24. Latent profile analysis (LPA) and multinomial logistic regression were conducted using Mplus 8.3 [33]. The Harman single-factor method was utilized to assess common method bias (CMB) [34] and the first factor accounting for 28.31% of the variance. This value, being below the 40% threshold, indicates that CMB is unlikely in this study [34]. To identify and characterize the ASD profiles of college students using LPA, individual response probabilities to specific ASD-related questions were utilized. Several LPA models with differing profile counts were evaluated to enhance model fit and ascertain the suitable number of profiles. The evaluation of model fit was based on several criteria as follows [35,36,37].

Specifically, the Akaike Information Criterion (AIC) measures the relative quality of statistical models by balancing model fit and complexity through penalizing the number of estimated parameters. The Bayesian Information Criterion (BIC) also evaluates model fit but imposes a stricter penalty for the number of parameters, making it more conservative than the AIC. The sample-size-adjusted Bayesian Information Criterion (aBIC) further refines the BIC by accounting for sample size, offering a more accurate assessment in smaller samples. The bootstrapped likelihood ratio test (BLRT) assesses model improvements by comparing nested models and using bootstrap sampling to estimate the test statistic’s distribution. Lastly, the Lo–Mendell–Rubin adjusted likelihood ratio test (LMRT) tests the fit improvement between models differing by one class, providing a *p*-value to determine the significance of adding an additional class [38]. Preferred models are indicated by lower AIC, BIC, or aBIC values. The entropy value represents the precision of the classification, with values above 0.80 indicating an acceptable level [39]. The minimum estimated profile proportion was defined as 5% of the overall sample size [40]. A multinomial logistic regression analysis was performed to explore the association between the identified latent profiles and demographic variables in mixture modeling, employing a three-step approach [41]. Furthermore, the associations between identified latent profiles and depressive symptoms, anxiety, and life satisfaction were evaluated through the BCH approach [42].

## 3. Results

### 3.1. Latent Profile Analysis of ASD

Table 1 presents the fit indices for one- to four-profile analysis solutions of students’ ASD symptoms. Lower values of the AIC, BIC, and aBIC indicate better model fit, while higher entropy values suggest clearer delineation between profiles. The four-profile solution exhibited the lowest AIC (8128.61), BIC (8245.64), and aBIC (8172.59), with a high entropy value (0.868), indicating the best fit and good classification quality. Additionally, BLRT and LMRT *p*-values were significant for all models (*p* < 0.001 for BLRT; *p* < 0.011 for LMRT), supporting the preference for more complex models. However, the fourth profile’s small prevalence (0.016), which is below 5%, suggests possible misfit and unsuitability, making the three-profile solution a more practical choice despite the superior statistical fit of the four-profile solution. Consequently, the results highlight the balance between statistical fit and practical application in selecting the appropriate model. Therefore, we chose the three-profile solution.

**Figure 2:**
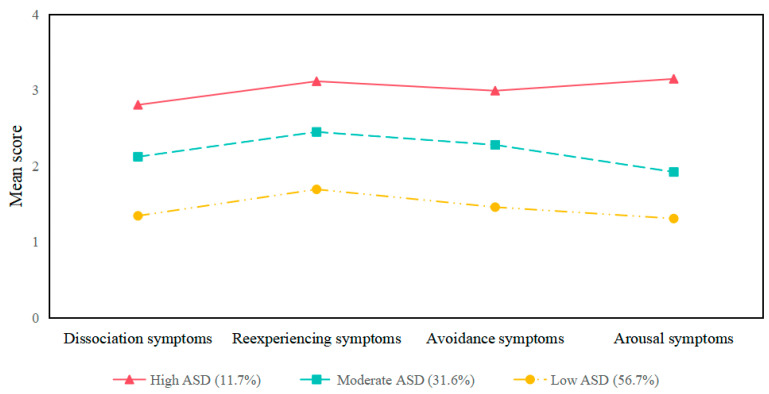
Latent profile plot of college students’ ASD symptoms.

The LPA results, depicted in Table 2 and Figure 2, identify three distinct profiles of student ASD symptom severity during the early COVID-19 outbreak. Each profile is visually represented based on the mean scores. The high ASD profile (11.7%) exhibited the most severe symptoms across four dimensions: dissociation, reexperiencing, avoidance, and arousal. The moderate ASD profile (31.6%) showed intermediate levels of symptoms in these dimensions. The low ASD profile (56.7%) had the least severe symptoms, indicating minimal stress responses. This analysis provides a detailed understanding of how ASD symptoms manifest in different severity levels among college students during the pandemic.

The results of the latent classification probabilities are presented in Table 3, demonstrating the accuracy of the model in classifying individuals into high, moderate, or low ASD profiles. The probability of individuals in the high ASD profile being correctly classified is 0.947, indicating a high level of precision for this group. For the moderate ASD profile, the classification probability is 0.877, reflecting strong accuracy, though slightly lower than that of the high ASD profile. The low ASD profile has the highest classification probability at 0.952, signifying excellent effectiveness in identifying members of this profile. These results underscore the robustness of the latent profile analysis in accurately categorizing individuals based on their ASD symptom severity.

### 3.2. Predictors of ASD Profiles in Student Characteristics

We examined the impact of individual student characteristics on profile membership classification using multinomial logistic regression with the R3STEP approach. Table 4 presents the multinomial logistic regression results predicting ASD profile membership across three categories: high, moderate, and low ASD. The results indicate that gender is a significant predictor, with females more likely to be in the high ASD group (OR = 1.86, *p* < 0.01) and in the moderate ASD group (OR = 2.03, *p* < 0.01) compared to in the low ASD group. Grade does not significantly predict ASD profile membership, as evidenced by non-significant coefficients (OR = 0.95, *p* > 0.05). Ethnicity is a significant predictor, with minority individuals more likely to belong to the high ASD group (OR = 1.55, *p* < 0.05) and to the moderate ASD group (OR = 1.60, *p* < 0.01). Socioeconomic status (SES, 1 = low, 5 = high) also significantly influences ASD profile membership, with a higher SES associated with lower odds of being in the high ASD group (OR = 0.71, *p* < 0.05) and in the moderate ASD group (OR = 0.76, *p* < 0.05). Similarly, self-rated health (1 = very poor, 5 = very good) is a significant predictor, with poorer self-rated health associated with higher odds of being in both the high ASD group (OR = 0.60, *p* < 0.001) and in the moderate ASD group (OR = 0.43, *p* < 0.001). Finally, higher perceived social support significantly decreases the likelihood of being in the moderate ASD profile compared to in the low ASD profile (OR = 0.43, *p* < 0.05).

### 3.3. Differences in Mental Health-Related Outcomes Among ASD Profiles

Table 5 presents the chi-square analysis results for differences in mental health across three ASD profiles using the BCH procedure. Cramér’s V was used to determine the effect sizes for the chi-square analyses [43,44]. The results indicate significant differences in anxiety levels across the profiles, χ^2^(2) = 371.60, *p* < 0.001. For anxiety, the effect size (Cramér’s V = 0.39) indicated a medium effect. Similarly, depression levels significantly differ among the profiles, χ^2^(2) = 194.54, *p* < 0.001. For depression, the effect size (Cramér’s V = 0.28) indicated a small to medium effect. Life satisfaction also shows significant variation across the profiles, χ^2^(2) = 15.27, *p* < 0.001. For life satisfaction, the effect size (Cramér’s V = 0.08) indicated a small effect.

## 4. Discussion

This research was the initial effort to identify latent profiles of college students exhibiting multiple ASD symptoms; investigate the related sociodemographic factors and levels of social support for each profile; and evaluate the connections between profile membership and anxiety, depression, and life satisfaction. Three distinct profiles were identified: high ASD, moderate ASD, and low ASD. Our findings provide valuable insights into understanding the different ASD latent profiles and identifying high-risk subgroups that are more likely to experience lower life satisfaction and increased anxiety and depressive symptoms. This study highlights the importance of targeted interventions aimed at these specific high-risk groups to enhance their mental health outcomes. This study further extends existing research by (1) revealing the ASD symptom profiles among university students during the COVID-19 pandemic and (2) enriching the understanding of antecedent and consequent variables associated with ASD symptom profiles.

Our research identified three distinct subtypes of ASD, consistent with previous findings [16,17,18]. This indicates that there are notable similarities in ASD profiles across different studies that have also employed person-centered analyses. Specifically, our study reinforces the robustness of person-centered methods in identifying heterogeneous ASD symptom profiles. These profiles—high ASD (11.7%), moderate ASD (31.6%), and low ASD (56.7%)—reveal varying levels of symptoms across four dimensions: dissociation, reexperiencing, avoidance, and arousal. Understanding these profiles provides valuable insights into the heterogeneity of ASD symptoms and their impacts on mental health during a global pandemic. The high ASD profile, characterized by the most severe symptoms across all dimensions, suggests that individuals in this group may experience higher exposure to multiple stressors, such as COVID-19-induced uncertainty, isolation, and quarantine [45], and possess insufficient coping resources [46]. The moderate ASD profile, with intermediate symptom levels, indicates a partial activation of stress responses. The low ASD profile, representing the majority of students (56.7%), exhibits minimal stress responses, suggesting effective coping strategies or lower exposure to stressors.

The multinomial logistic regression analysis reveals several key factors predicting ASD profile membership among undergraduates during COVID-19. Gender significantly predicts ASD profiles, with females tending to be in the high and moderate ASD groups compared to the low ASD group, consistent with research showing females exhibit higher stress and anxiety in response to stress-related disorders due to sex hormones [47]. In addition, women demonstrate greater sensitivity to negative emotional stimuli [48]. Recent meta-analytic findings emphasize that masculine traits significantly contribute to emotional health, whereas feminine traits do not exhibit the same level of protective influence [49]. Ethnicity is also significant, with minority students more likely to be in higher ASD groups, reflecting widening mental health disparities between ethnic groups [50]. Kamal et al. found that in the U.S., sexual and gender minority young adults experienced disproportionately higher psychiatric impacts from the pandemic, beyond what could be explained by traditional minority stress factors like family support and discrimination [51]. A higher SES is linked to lower odds of high and moderate ASD profiles, suggesting that greater resources and social support associated with a higher SES help buffer against acute stress [52]. Yoshioka et al. reported that in Japan, factors like economic instability, caregiving burdens, domestic violence, and fear of COVID-19 were strongly linked to serious psychological distress, particularly among young women [53]. Poor self-rated health is strongly associated with higher ASD profiles, aligning with findings that negative health perceptions increase psychological distress [54]. Additionally, higher perceived social support decreases the likelihood of being in the moderate ASD profile, supporting the stress-buffering model which posits that social support mitigates the impact of stress by providing essential resources [55]. These results underline the necessity of targeted interventions considering these demographic and psychosocial factors to support college students’ mental health.

The chi-square analysis results reveal significant differences in mental health outcomes—anxiety, depression, and life satisfaction—across the three identified ASD profiles. These findings offer valuable insights into the psychological impacts of ASD symptom severity among college students. Significant differences in anxiety levels were observed among the ASD profiles, aligning with previous research showing that higher ASD symptom severity correlates with elevated anxiety levels [2]. The transactional model of stress and coping (TMSC) suggests that severe stress responses lead to heightened anxiety due to ineffective coping strategies and perceived threats [46]. Depression levels also significantly differed among the profiles, consistent with the diathesis–stress model which posits that stress can trigger depressive symptoms, particularly in vulnerable individuals [56]. Life satisfaction varied significantly across profiles but with a smaller effect size. While ASD symptom severity impacts life satisfaction, the effect is less pronounced compared to anxiety and depression. These findings highlight the importance of considering specific ASD symptom patterns when addressing the mental health needs of university students, especially during crises like the COVID-19 pandemic.

### 4.1. Implications

This research constitutes the initial effort to categorize among college students during the early stages of the COVID-19 pandemic. Theoretically, this research expands the application and explanatory power of the TMSC and the stress-buffering model within the context of ASD during a pandemic [46,57]. Methodologically, the identified ASD profiles underscore the importance of employing person-centered approaches in psychological assessments and interventions. This method enables a detailed comprehension of individual differences in stress responses and mental health outcomes, which can lead to more tailored and effective interventions. By adopting these person-centered methods, this study contributes significantly to both theoretical and practical advancements in the field of mental health, particularly in understanding and addressing the complex impacts of acute stress among college students during unprecedented times. The implications of this study also extend to policy-making and resource allocation within educational institutions. By recognizing the distinct profiles of ASD, universities can develop more effective support systems that satisfy the specific needs of different student groups. Coulaud et al. highlighted that in Canada and France, young adults faced significant unmet mental health service (MHS) needs during the pandemic, with over half of those seeking services unable to access them, especially men and those who lost income due to COVID-19 [58]. Policies that facilitate access to mental health resources, reduce isolation, and provide clear communication about health and safety measures can alleviate some of the stressors associated with the pandemic [59].

### 4.2. Limitations and Directions for Future Research

Several limitations in this study should be acknowledged. First, the reliance on self-reported measures of ASD and related variables may have introduced social desirability bias, potentially leading to underreporting of ASD symptoms and overreporting of mental health difficulties. Second, the study participants were restricted to Chinese undergraduates, excluding those from other countries or educational stages, which could impact the applicability of the findings. Subsequent research could incorporate a broader and more varied sample to improve the generalizability the results. Third, the potential predictive role of other personal factors, such as personality traits, in shaping specific ASD profiles is also worth noting. These factors may play a crucial role in influencing stress-coping mechanisms, warranting further exploration in future research. Additionally, the cross-sectional design of this study precludes causal inferences about the relationship between ASD profiles and anxiety, depression, and life satisfaction. Longitudinal studies are recommended to further explore these associations. Similarly, investigating transitions among ASD profiles over time and conducting prospective studies to determine if latent ASD profiles predict mental health outcomes is encouraged. Finally, cognitive behavior therapy (CBT) is the preferred treatment for ASD. Future research should investigate the efficacy of CBT across different ASD subgroups to determine its varying impacts.

## 5. Conclusions

In conclusion, our findings highlight the varying impacts of ASD symptom severity on mental health outcomes among undergraduates during the pandemic. The significant differences in anxiety, depression, and life satisfaction across the ASD profiles emphasize the necessity for specialized interventions to improve students’ mental health. By leveraging theoretical frameworks and empirical evidence, mental health professionals can develop effective strategies to address the unique needs of each ASD profile, ultimately enhancing the well-being of college students during this worldwide pandemic.

## Figures and Tables

**Table 1:** Fit indices for 1–4-profile analysis solutions of students’ ASD symptoms.

Profiles Number	AIC	BIC	aBIC	Entropy	BLRT (*p*)	LMRT (*p*)	Profile Prevalence
1	10,549.96	10,590.67	10,565.26	—	—	—	—
2	8831.80	8897.95	8856.66	0.877	<0.001	<0.001	0.747/0.253
**3**	**8331.89**	**8423.48**	**8366.30**	**0.839**	**<0.001**	**<0.001**	**0.567/0.316/0.117**
4	8128.61	8245.64	8172.59	0.868	<0.001	0.011	0.551/0.303/0.130/0.016

Note. Boldface type indicates the selected model.

**Table 2:** Descriptive results and membership percentages across three ASD profiles.

ASD Symptoms	High ASD Profile (11.7%)		Moderate ASD Profile (31.6%)		Low ASD Profile (56.7%)	
	**M**	***SD***	**M**	***SD***	**M**	***SD***
Dissociation	2.81	0.51	2.12	0.51	1.34	0.51
Reexperiencing	3.12	0.54	2.45	0.54	1.69	0.54
Avoidance	2.99	0.52	2.28	0.52	1.46	0.52
Arousal	3.15	0.36	1.92	0.36	1.31	0.36

Note. *N* = 1198; ASD = acute stress disorder.

**Table 3:** The results of the latent classification probabilities.

Profile Type	High ASD Profile	Moderate ASD Profile	Low ASD Profile
High ASD profile	0.947	0.053	<0.001
Moderate ASD profile	0.016	0.877	0.106
Low ASD profile	<0.001	0.048	0.952

Note. *N* = 1198; ASD = acute stress disorder.

**Table 4:** Results of multinomial logistic regression.

	High vs. Low ASD		Moderate vs. Low ASD	
	***B***	**OR**	***B***	**OR**
Gender	0.71 **	1.86 *	0.62 **	2.03 *
Age	0.05	1.04	−0.01	0.99
Grade	−0.01	0.99	−0.05	0.95
Ethnicity	0.44 *	1.55	0.47 **	1.60 *
SES	−0.34 *	0.71 *	−0.27 *	0.76 **
Self-rated health	−0.84 ***	0.60 ***	−0.50 ***	0.43 ***
Perceived social support	0.07	1.07	−0.18 *	0.83 *

Note. *N* = 1198, gender (0 = male, 1 = female), ASD = acute stress disorder, ethnicity (0 = Han, 1 = Minority), OR = odds ratio, reference group = low ASD profile, * *p* < 0.05, ** *p* < 0.01, *** *p* < 0.001.

**Table 5:** Chi-square analysis results for differences in mental health across three ASD profiles.

	High ASD Profile		Moderate ASD Profile		Low ASD Profile		Overall χ^2^ Test Value (df)	Effect Size (Cramér’s *V*)
	**M**	***SE***	**M**	***SE***	**M**	***SE***		
Anxiety	2.03	0.06	1.56	0.02	1.18	0.01	χ^2^(2) = 371.60 ***	0.39
Depression	1.81	0.06	1.39	0.03	1.12	0.01	χ^2^(2) = 194.54 ***	0.28
Life satisfaction	3.18	0.11	3.20	0.07	3.53	0.05	χ^2^(2) = 15.27 ***	0.08

Note. *N* = 1198, *** *p* < 0.001, ASD = acute stress disorder.

## Data Availability

Data can be available on reasonable request from the authors.
